# Textbook of Psychiatry

**Published:** 1907-05

**Authors:** 


### BOOKS AND AUTHORS.

**Textbook of Psychiatry**. A Psychological Study of Insanity for Practitioners and Students. By **E. Mendel**, M.D., A. O. Professo[illegible] in the University of Berlin. Authorised translation. Edited and enlarged by **William C. Krauss**, M.D., Buffalo, N. Y., President Board of Managers Buffalo State Hospital for Insane; Medical Superintendent Providence Retreat for Insane; Neurologist to Buffalo General, Erie County, German, Emergency Hospitals, etc.; Member of the American Neurological Association. 311 pages. Crown Octavo. Philadelphia: F. A. Davis Company. (Price, Extra Cloth, $2.00 net).

Professor Mendel has been identified so prominently with the teaching of psychiatry in Germany for the past thirty years, that a textbook which contains the result of his studies is a welcome addition to medical literature. No psychiatrist from America has ever visited Professor Mendel's clinic without coming away impressed by his scientific method and great attainment in this subject. This work is eminently the result of clinical observation. It abounds in notes which illustrate the various types of insanity. His classification of insanity into mania, melancholia, circular psychoses, acute dementia, epileptic psychoses, hysterical psychoses, choreic psychoses, intoxicant's psychoses, psychoses due to functional disturbances of the thyroid gland, psychoses of infection, psychoses from autointoxication, psychoses from organic poisons, psychoses from inorganic poisons, organic psychoses due to diseases of the brain cortex, cerebral dementia, arteriosclerotic psychoses, syphilitic psychoses, psychoses after apoplectic attacks, psychoses from brain tumors, and psychoses from trauma, is clear and reasonable as the author shows, and it will if followed enable the physician to make the diagnosis in a great majority of cases.

In studying this work we note that great attention is paid to the study and classification of hallucinations, delusions, disturbances of the feeling of judgment, disturbances of consciousness, such as stupor and mania. There is a series of plates illustrating the various stigmata of degeneration, which consider the configuration of the scalp, all deformities of the ear, the description of the eye, all degenerations and deformities of the palate, and abnormalities of the teeth. The directions for the examination of patients for the purpose of making a diagnosis in the supplement, are very complete, and if followed, the beginner can hardly fail to reach a decision as to the form of the disease.

Dr. Krauss is to be congratulated upon his translation, which has placed before American readers so perfectly this important work in psychiatry. Dr. Krauss's enlargement on the chapters of degeneracy and heredity have added materially to the value of the book. We speak for it a wide reading.

